# High threshold of β1 integrin inhibition required to block collagen I-induced membrane type-1 matrix metalloproteinase (MT1-MMP) activation of matrix metalloproteinase 2 (MMP-2)

**DOI:** 10.1186/s12935-014-0099-3

**Published:** 2014-10-03

**Authors:** Kulrut Borrirukwanit, Prasit Pavasant, Tony Blick, Marc A Lafleur, Erik W Thompson

**Affiliations:** Department of Nursing, Phetchabun Hospital, Phetchabun, Thailand; Invasion and Metastasis Unit, St. Vincent’s Institute, Fitzroy, Victoria 3065 Australia; Department of Anatomy, Faculty of Dentistry, Chulalongkorn University, Chulalongkorn, Thailand; Department of Surgery, St Vincent’s Hospital, University of Melbourne, Fitzroy, Victoria Australia; Institute of Health and Biomedical Innovation and School of Biomedical Sciences, Queensland University of Technology, Kelvin Grove, Queensland Australia

**Keywords:** β1 Integrin, Matrix metalloproteinase, MMP-2 activation, Type I collagen, Invasion and metastasis

## Abstract

**Background:**

Matrix metalloproteinase-2 (MMP-2) is an endopeptidase that facilitates extracellular matrix remodeling and molecular regulation, and is implicated in tumor metastasis. Type I collagen (Col I) regulates the activation of MMP-2 through both transcriptional and post-transcriptional means; however gaps remain in our understanding of the involvement of collagen-binding β1 integrins in collagen-stimulated MMP-2 activation.

**Methods:**

Three β1 integrin siRNAs were used to elucidate the involvement of β1 integrins in the Col I-induced MMP-2 activation mechanism. β1 integrin knockdown was analyzed by quantitative RT-PCR, Western Blot and FACS analysis. Adhesion assay and collagen gel contraction were used to test the biological effects of β1 integrin abrogation. MMP-2 activation levels were monitored by gelatin zymography.

**Results:**

All three β1 integrin siRNAs were efficient at β1 integrin knockdown and FACS analysis revealed commensurate reductions of integrins α2 and α3, which are heterodimeric partners of β1, but not αV, which is not. All three β1 integrin siRNAs inhibited adhesion and collagen gel contraction, however only the siRNA showing the greatest magnitude of β1 knockdown inhibited Col I-induced MMP-2 activation and reduced the accompanying upregulation of MT1-MMP, suggesting a dose response threshold effect. Re-transfection with codon-swapped β1 integrin overcame the reduction in MMP-2 activation induced by Col-1, confirming the β1 integrin target specificity. MMP-2 activation induced by TPA or Concanavalin A (Con A) was not inhibited by β1 integrin siRNA knockdown.

**Conclusion:**

Together, the data reveals that strong abrogation of β1 integrin is required to block MMP-2 activation induced by Col I, which may have implications for the therapeutic targeting of β1 integrin.

## Background

Integrins are a family of transmembrane adhesion receptors that mediate cell-matrix and cell-cell interactions [1]. β1 integrin is one of the β subunits that mechanically link extracellular matrix (ECM) ligands to the cell by triggering formation of intracellular cytoskeletal scaffolds that facilitate interactions between signaling proteins [2]. Importantly, β1 integrin signaling has been shown to mediate diverse roles in cancer progression including invasion, migration and metastasis [3-5]. The importance of proteolytic enzymes in facilitating invasive tumor growth has been recognized, with major contributions by matrix metalloproteinases (MMPs) [6-8].

The MMP family consists of more than 26 endopeptidases and has a clear connection to ECM degradation and cancer cell invasion. It is increasingly implicated in the proteolysis of important cell regulatory molecules, including cytokines and growth factors, and their receptors and/or inhibitors [9]. Although several MMPs have been associated with tumor progression, particular attention has been focused on MMP-2 and membrane type-1 MMP (MT1-MMP) [10,11]. MT1-MMP has a large number of substrates including ECM (e.g. collagens, fibronectin, laminins) and non-ECM molecules, and is a potent modifier of the tissue microenvironment. Its expression is closely associated with tumour growth and invasion in animal models, and cancer progression in patients [12-14].

Activation of pro-MMP-2 proceeds on the cell surface and is mediated by a tri-molecular complex of MT1-MMP, TIMP-2 and MMP-2 [15,16]. Collagen type I (Col I) has been shown to stimulate MT1-MMP-mediated MMP-2-activation in several cell types [17-22]. Col I can cause activation of MMP-2 through increasing MT1-MMP mRNA and protein levels (transcriptional response). However, an alternate pathway has been observed in which Col I increases the activity of pre-existing MT1-MMP molecules in order to activate MMP-2 [23]. We previously demonstrated that Col I induces MT1-MMP-mediated MMP-2 activation by blocking MT1-MMP internalization, thereby stimulating MMP-2 activation [24]. However, previous studies have indicated that cellular interaction with Col I is mediated largely through integrin α1β1, α2β1 and α3β1 receptors [25-27], and cross linking of integrin β1 could activate MMP-2 in ovarian carcinoma cells, suggesting direct involvement of integrin signaling in MMP-2 activation [21,28].

Involvement of integrin β1 activation has been demonstrated in MT1-MMP effects on skeletal stem cell commitment [29], and Mori *et al.* have implicated the transmembrane/cytoplasmic domains of MT1-MMP in mammary branching morphogenesis via direct binding to integrin β1 [30]. Despite this clear implication of β1 integrin in MT1-MMP functionality, the involvement of β1 integrin in MMP-2 activation in response to Col I stimulation is still not precisely understood, with a recent study suggesting a direct interaction between MT1-MMP and Col I leading to MT1-MMP stabilization in an integrin-independent manner [31]. Our study further investigated β1 integrin involvement in MMP-2 activation in response to Col I stimulation in breast cancer cells, and found it played an important role in this process.

## Materials and methods

### Plasmids, siRNA, antibodies and reagents

MCF-7-MT1 cells: Stable transfection of MT1-MMP into MCF-7 cells previously transfected with β-galactosidase was previously described [32,33]. The pcDNA-β1 vector was constructed by cloning human β1 integrin cDNA into pcDNA3.1. A codon-swapped mutant form of the human β1 integrin (β1-Mt) was generated by the QuickChange site-directed mutagenesis kit (Invitrogen, Melbourne, Australia). Two synthetic oligonucleotide primers were designed to generate 7 point mutations in the β1/6 integrin siRNA recognition site: TGCTGATATGGAAACTACTTATGATTATACACGACAGAAGGGAGT and ACTCCCTTCTGTCGTGTAT AATCATAAGTAGTTTCCATATCAGCA.

Three β1-integrin siRNAs (Pro-Oligo, Australia) were found active for β1 integrin knockdown: β1-integrin siRNA#4 (β1/4), AATTAGCATAACTTCAAATAA, β1-integrin siRNA#5 (β1/5), AATGGCTTAATTTGTGGAGGA, and β1-integrin siRNA#6 (β1/6), AAGCTTTTAATG ATAATTCAT.

Antibodies against the following proteins were purchased from Chemicon (Boronia, Australia): integrin β1 (AB2510), αV (L320), α1 (MAB19732) α3 (MAB19522) and MT1-MMP (AB815). Antibody toward α2 integrin (Ak7) was a kind gift from Dr. Michael Berndt (Monash University, Melbourne) and a pan-actin antibody was from Biosource (Camarillo, CA). Con A, TPA (12-O-tetradecanoylphorbol-13-acetate), vitronectin (VN), fibronectin (FN) and laminin-1 (LM) were from Sigma (Castle Hill, Australia). Recombinant full-length pro-MMP-2 expressed in a vaccinia virus system (rMMP-2) was a kind gift from Dr. Rafael Fridman (Wayne State University, Detroit, MI). Col I (Vitrogen 100) was obtained from Cohesion (Palo Alto, CA). Alexa Fluor 568-conjugated goat anti-mouse secondary antibody and Alexa Fluor 488-conjugated donkey anti-rabbit secondary antibody were obtained from Molecular Probes (Eugene, OR).

### Cell culture and transfection

MCF-7-MT1 and MDA-MB-231 cells were maintained in Dulbecco’s Modified Eagle’s Medium (DMEM; Life Technologies, Auckland, New Zealand) with 10% fetal bovine serum (FBS; JRH Biosciences, Lenexa, KS). SiRNA transfections were performed using Lipofectamine™ 2000 (Invitrogen, Life Technologies). Plasmid transfections were performed using Fugene6 reagent (Roche, Mannheim, Germany) with 2 μg of plasmid DNA. For co-transfection of human β1 integrin and β1 integrin siRNAs, MCF-7-MT1 and MDA-MB-231 cells were transfected with β1/6-integrin siRNA using Lipofectamine™ 2000 as described above. The siRNA-transfected cells were subsequently transfected with either wild-type or mutant β1 integrin as indicated using Fugene6 with 2 μg plasmid DNA. Recombinant full-length pro-MMP-2 was added at 100 ng/ml. At the appropriate time point, the conditioned media and cell lysates were collected.

### DNA synthesis and quantitative PCR

cDNA was prepared from 100 ng of total RNA isolated with the Qiagen RNeasy mini-column kit (Qiagen, GmbH, Germany) using Superscript II reverse transcriptase (Invitrogen) and random primers. Human ribosomal protein L32 mRNA was used as a housekeeping gene for normalization [34]. qRT-PCR was performed on an ABI Prism 5700 Sequence Detection System (Perkin-Elmer Applied Biosystems, Australia) with cDNA generated from an equivalent of 2 ng of RNA as previously described [24]. The primer sequences used were for L32: CAGGGTTCGTAGAAGATTCAAGGG and CTTGGAGGAAACATTGTGAGCGATC; α1 integrin: GCTGACCAGTCAGCAGCTTCATTT and CTCCAGAAGAAGCAGTAGCAGAGTTT; α2 integrin: GACCTATCCACTGCCACATGTGAAAAA and CCACAGAGGACCACATGTGAGAAAA; α3 integrin: CGCAGGTGGGCGCCTATTTT and GGCACCCCCTACTTCCTCTTT; αV integrin: GGCAGTGC CATAGCTCCTTT and CCCACTGCCCTTCAAGGGATTT; β1 integrin: GACTGATCAGTTCAGTTTGCTGT GTGTTT and CCCTGCTTGTATACATTCTCCACATGATTT. Reaction conditions were 95°C for 10 minutes followed by 50 cycles of 95°C for 15 s and 60°C for 1 minute. The difference in average cycle threshold (dCT) between L32 (housekeeping) and the gene of interest was determined from quadruplicate readings for each sample.

### Flow cytometry

Cells were trypsinised and resuspended in FACS buffer (2% FCS in PBS, 0.02% sodium azide) at 1×10^6^ cells/ml, then incubated with 10 μg/ml antibodies to integrins α1, α2, α3, αV, and β1. Cells were then washed and incubated with FITC-conjugated goat anti-mouse or swine anti-rabbit IgG (Dako) and analyzed on a FACScan cytometer (Becton-Dickinson, Mountain View, CA).

### Immunofluorescence

Cells were plated on teflon printed glass slides (Electron Microscopy Sciences, Ft. Washington PA) and fixed with 3% paraformaldehyde/PBS then blocked with 5% BSA/0.25% Triton X-100. Cells were then washed in PBS and incubated with primary antibodies toward β1 integrin (AB2511) or MT1-MMP (AB815). Cells were washed and probed with a donkey anti-rabbit Alexa Fluor 488-conjugated antibody for β1 integrin or an Alexa Fluor 568 secondary antibody for MT1-MMP. Nuclei were then stained with propidium iodide (0.25 mg/ml), then the cells were washed and mounted using fluorescent mounting medium (Dako) and viewed by confocal microscopy (BioRad MRC 1024, Hemel Hempstead, UK).

### Cell lysates and Western blot

Cells were lysed with 10 mM Tris–HCl pH 7.6, 10 mM NaCl, 3 mM MgCl_2_ and 1% Nonidet P-40 containing Protease Inhibitors (Roche), then cleared by centrifugation at 6,000 rpm for 10 minutes. Protein concentrations were determined with a BCA protein quantification assay (Pierce, Rockford IL). Equal amounts of lysates were electrophoresed with 10% SDS-PAGE under reducing conditions (100 mM DTT, Sigma). After electrophoresis, proteins were transferred to PVDF membranes and blocked with 5% skim milk. Blots were incubated with primary antibodies overnight and then probed with an HRP-conjugated secondary antibody (Pierce). Detection was performed using the ECL + Plus system (Pierce).

### Conditioned medium isolation and gelatin zymography

Cells were plated for near confluence at 37°C overnight. After each treatment, cells were washed twice with serum-free medium (SFM: unsupplemented DMEM), then replaced with fresh SFM. Recombinant full-length pro-MMP-2 (kindly, provided by Dr. Rafael Fridman, Wayne State University, Detroit, MI, USA) was added at 100 ng/ml concentration. At the appropriate time point, the conditioned medium was collected, transferred to a microcentrifuge tube and centrifuged at 6000 rpm for 10 minutes at 4°C. MMP-2 activation was analyzed by gelatin zymography using 5% polyacrylamide stacking gel and 10% polyacrylamide resolving gel (Bio-Rad Co., Richmond, VA) containing 1 mg/ml gelatin (BDH Laboratory Supplies, Poole, UK). Equal amounts of conditioned media were mixed with SDS sample buffer under non-reducing conditions. After electrophoresis, gels were washed with 50 mM Tris–HCl pH 7.5, 5 mM CaCl_2_ and 2.5% Triton X-100 and then incubated in 50 mM Tris–HCl pH 7.5, 5 mM CaCl_2_ at 37°C overnight. Gels were stained with 0.25% Coomassie Brilliant Blue (R-250) dye in 10% acetic acid and 10% isopropanol. Semiquantitative densitometry was performed using the Image J 1.46 program (NIH). Data is expressed as percent of area.

### Collagen gel contraction assay

Collagen gels were made from Col I (Vitrogen 100, 3 mg/ml), neutralized with 1 M sodium hydroxide, pH 8.0 and then diluted in PBS to a final concentration of 2 mg/ml of Col I in 10 mM of sodium hydroxide. Cells were resuspended such that one part of the cell suspension was mixed with nine parts of the collagen solution, to give a final concentration of 4×10^5^ cell/ml. A total of 500 μl of cell-collagen mixture was added to 24 well plates and gels were allowed to polymerize for 60 min at 37°C. In order to release the gels, a yellow tip was inserted around and underneath the gels. The relaxed, floating gels were immersed with 1 ml medium and incubated at 37°C. The gel diameters were measured daily for 5 days using an inverted microscope. Collagen gel contraction is indicated as the decrease in gel area expressed as a percentage. Each experiment was performed in triplicate and with 3 independent experiments. Statistical analysis was performed by Student’s t-test.

### Adhesion assay

Col I, fibronectin and vitronectin (0.5 ug/ml, 100 uL/well) were coated on 96-well plates at 37°C for 60 minutes and the wells were then washed and blocked in 3% BSA. Cells were detached from culture by Versene, resuspended at 2.5× 10^5^ cells/ml, and incubated at 37°C for 60 minutes to allow recovery of cell surface proteins. Cells (100 μl; 2.5× 10^4^ cells) were added to the wells and incubated at 37°C for 60 minutes. The adherent cells were stained with 0.5% Crystal Violet in 25% methanol for 5 minutes. Wells were then washed, dried and solubilization of the Crystal Violet was performed with 0.1 M sodium citrate containing 50% ethanol. Absorbance was measured at 540 nm (Power Wave X, Bio-Tek Instruments, Inc., Winooski, VT). Each experiment was performed in triplicate and 3 independent experiments were performed. Student’s t-test was used for statistical analysis.

## Results

### Knockdown of β1 integrin expression by siRNAs

**Figure 1:**
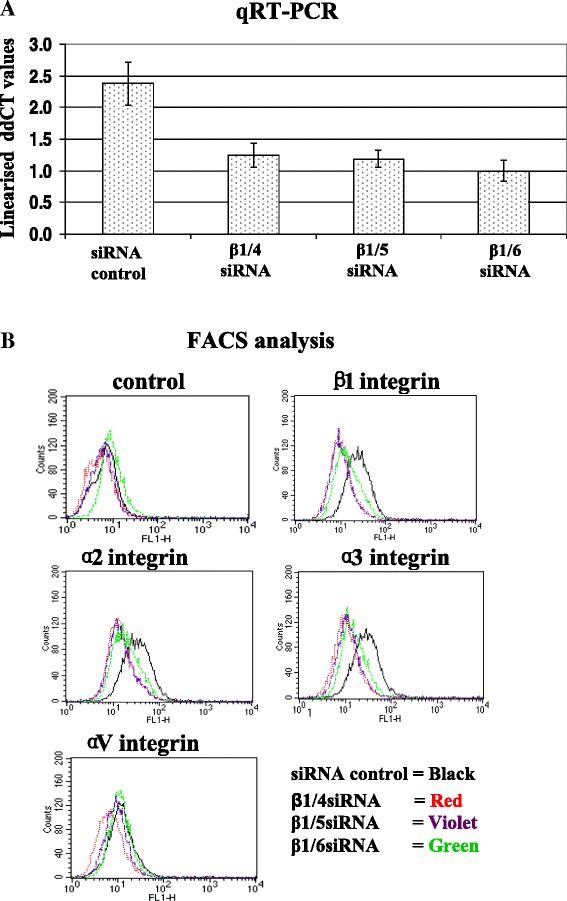
**Knockdown of β1 integrin expression by β1 siRNA in MCF-7-MT1 cells.** Integrin β1 and control siRNAs were transfected into MCF-7-MT1 cells. **(A)**: RNA was collected after 24 hours transfection and analyzed for β1 integrin expression by qRT-PCR. **(B)**: 72 hours after transfection, cells were analyzed by flow cytometry for α2, α3, αV and β1 integrin. Control panel depicts cells stained with secondary antibody alone.

β1 siRNAs β1/4, β1/5 and β1/6 were effective in knocking-down β1 integrin expression, however, β1/6 siRNA was more efficient than β1/4 and β1/5 in MCF-7-MT1 (Figure 1A) and MDA-MB-231 cells (data not shown). We hypothesized that β1 integrin knockdown would also affect the cell surface expression of the α subunit heterodimer partners. Integrin α1β1, α2β1, α10β1 and α11β1 are the major collagen-binding integrin receptors [35,36]. MCF-7-MT1 cells had very low levels of α1, and no detectable levels of α10 or α11 integrin mRNA (data not shown). FACS analysis for α2, α3, and αV integrins showed that all three siRNAs also caused reduced cell surface levels of α2 and α3 integrin subunits. MCF-7-MT1 cells had low levels αV integrin, and no effect of the β1 siRNAs on αV integrin levels was observed (Figure 1B).

### β1/6 siRNA demonstrated the most effective abrogation of β1 integrin biological function

**Figure 2:**
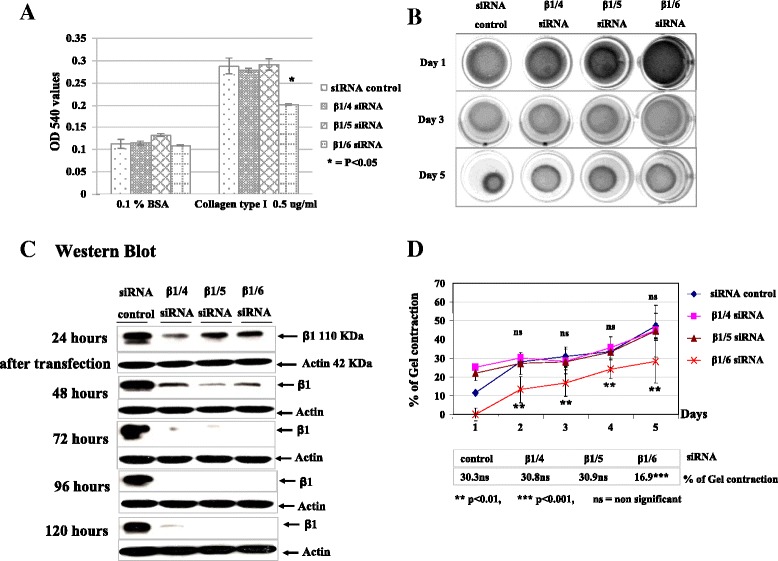
**Reduced functional activity in β1 integrin knockdown cells: (A): Adhesion assay of β1 integrin knockdown MCF-7-MT1 cells for Col I.** **(B)**: MDA-MB-231 cells were transfected with the indicated siRNAs. At appropriate time points, cell lysates were collected for analysis of β1 integrin levels by Western blot. **(C)**: Collagen gel contraction by MDA-MB-231 cells after transfection with β1/4, β1/5, β1/6 and control siRNAs. 24 hours after transfection, collagen gels were made at 2 mg/ml and the cells resuspended to a final concentration of 4 × 10^5^ cell/ml. Gels were polymerized and then the gels were released. The relaxed, floating gels were immersed in 1 ml medium with 10% FCS. The gel diameters were measured daily for 5 days. Collagen gel contractions were calculated as mean values in relation to the gel diameters before releasing, as shown in **(D)**. Collagen gel contractions were performed in triplicate independently and mean percent of collagen gel contraction was compared using Student’s t-test.

β1 integrin can bind multiple additional partners to form integrins in addition to the primary Col I receptors α1β1, α2β1, α10β1 and α11β1 [35,36]. Integrins α3β1, α6β1, α6β4 and α7β1 are major LM receptors [37]. Integrins α5β1 and α8β1 are major FN receptors. Integrin αIIbβ3 is an alternate FN receptor, while αvβ3 is specific for VN [36]. To examine the functional effects of inhibiting integrin β1 expression, adhesion analysis was performed with ECM substrates including VN, FN, and Col I after β1 integrin siRNA transfection. β1/6 integrin siRNA-treated cells showed significantly less adhesion (p < 0.05) to Col I (Figure 2A). Decreased adhesion to FN was observed for β1/4, β1/5 and β1/6 siRNAs compared to the control siRNA cells (p < 0.05, 0.05 and 0.01 respectively), and again β1/6 integrin siRNA showed a stronger effect than β1/4 and β1/5 (data not shown). None of the β1 siRNA treated cells showed any reduction in adhesion to VN (ligand for αvβ3), confirming that the β1 effects were specific (data not shown).

Collagen gel contraction was clearly observed with MDA-MB-231 cells and partial contraction was seen with MCF-7-MT1 cells. Collagen gel contraction appeared completely inhibited by β1/6 siRNA compared to β1/4 and β1/5 at Days 1, 3 and 5 in MDA-MB-231 cells (Figure 2C). All of the β1 integrin siRNA treated cells showed less collagen contraction than the siRNA control at Day 5 (Figure 2C). However, only β1/6 siRNA-treated cells showed significantly (p < 0.01) reduced ability to initiate collagen gel contraction on the first and all subsequent days (Figure 2D), while β1/4 and β1/5 integrin siRNAs initially failed contraction on the first day, but resumed contraction on the second and subsequent days. Consistent with the above results, only β1/6 integrin siRNA caused a significant reduction in collagen gel contraction (16.9%, p <0.001) compared to β1/4 and β1/5 (Figure 2D).

### Abrogation of β1 integrin expression suppressed Col I-induced MMP-2 activation via MT1-MMP up-regulation

**Figure 3:**
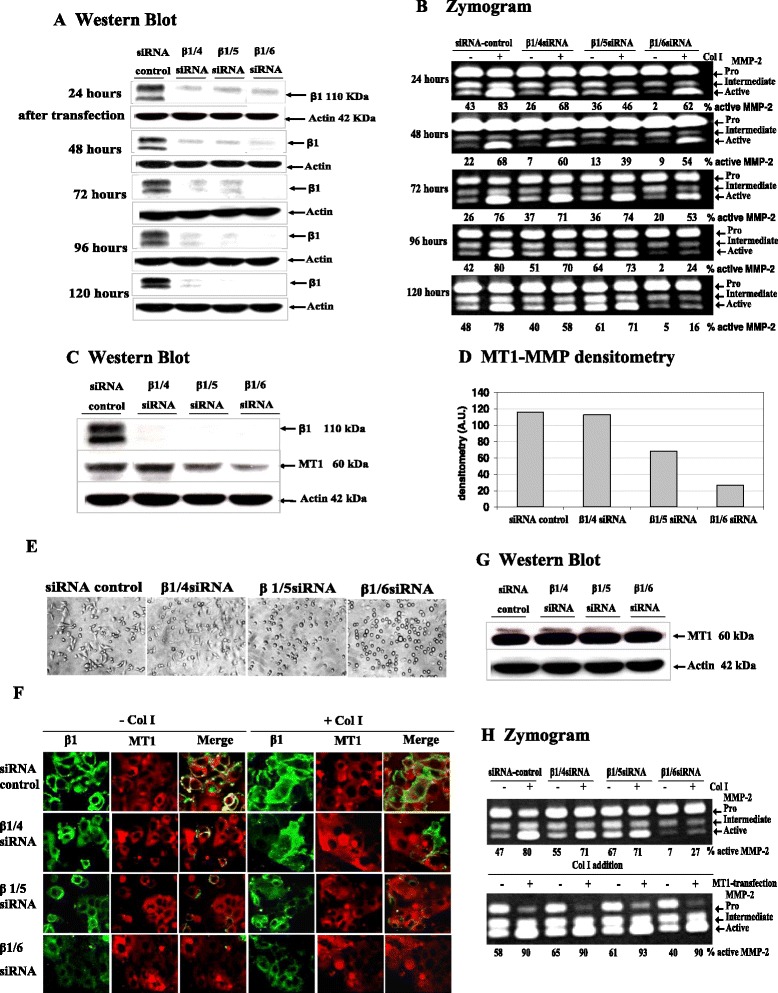
**Time course of β1 integrin knockdown and MMP-2 activation in MCF-7-MT1 cells. (A)**: MCF-7-MT1 cells were transfected with the indicated siRNAs. At the indicated time points, cell lysates were collected for analysis of β1 integrin levels by Western blot. **(B)**: Zymogram: MCF-7 cells were transfected with the indicated siRNAs. rMMP-2 was added at serial time points after β1 integrin siRNA transfection and cells were treated where indicated with Col I. Conditioned medium was collected and MMP-2 activation was assessed by zymography. Semiquantitative densitometry was performed and is expressed as percent area. **(C)**: MT1-MMP levels were reduced in β1 integrin - abrogated cells. MCF-7-MT1 cells were transfected with the indicated siRNAs, and protein levels of β1 integrin and MT1-MMP were examined by Western blot at 72 hours after transfection. **(D)**: MT1-MMP densitometry was analyzed and is expressed in arbitrary units. **(E)**: The indicated siRNAs were transfected into MCF-7-MT1 cells, incubated for 6 hours cells were then mounted and viewed by light microscopy. **(F)**: Immunofluorescence analysis for β1 integrin and MT1-MMP at the MCF-7-MT1 cell surface. After 72 hours transfection, cells were plated on Teflon printed glass slides and treated with Col I where indicated. Cells were subjected to immunofluorescence and then viewed by confocal microscopy. **(G)**: Ectopic MT1-MMP expression rescued suppression of Col I-induced MMP-2 activation by β1/6 integrin siRNA. MCF-7-MT1 cells were transfected with the indicated siRNAs, and subsequently transfected with MT1-MMP. Cells were then left untreated or treated with Col I. Efficiency of MT1-MMP inductions were determined by Western Blot. **(H)**: MMP-2 activation was analyzed by zymography. Semi-quantitative densitometry is expressed in percent of area.

In MCF-7-MT1 cells, Col I caused activation of MMP-2 despite MT1-MMP being under the control of a heterologous promoter. Furthermore, when these cells were treated with cycloheximide to block new protein synthesis, some reduction in MMP-2 activation was seen, but the MMP-2 activational response to Col I was still observed [32]. Thus, MCF-7-MT1 cells provide an ideal model for studying non-transcriptional regulation of Col I-induced MMP-2 activation. Again, integrin expression appeared completely abrogated by β1/6 siRNA compared to β1/4 and β1/5 at 72, 96 and 120 hours in MCF-MT1 cells (Figure 3A). All three β1 integrin siRNAs caused reduced MMP-2 activation. Both β1/4 and β1/5 integrin siRNAs reduced Col I-induced MMP-2 activation at 24 hours and showed sustained reduction until 48 hours after knockdown, after which Col I-induced MMP-2 activation was not evident (i.e. between 72 and 120 hours after knockdown). On the other hand, reduced MMP-2 activation with β1/6 integrin siRNA was seen at 72 hours after knockdown, and continued to decrease, again showing that β1/6 was more efficient than β1/4 and β1/5 in both MCF-7-MT1 (Figure 3B) and MDA-MB 231 cells (data not shown)

Col I-stimulated MMP-2 activation functions principally via MT1-MMP in fibroblasts, as no MMP-2 activation occurs in MT1-MMP-deficient mouse fibroblasts in response to Col I [38,39]. Since we found that abrogation of β1 integrin expression was able to suppress Col I-stimulated MMP-2 activation, the influence of β1 integrin on MT1-MMP activity was examined. Interestingly, β1/5 and β1/6 integrin siRNAs caused noticeably decreased MT1-MMP levels, however, the β1/6 integrin siRNA showed the strongest decrease, which corresponded to the lowest β1 integrin level compared to controls (siRNA control) (Figure 3C and D) and the highest suppression of MMP-2 activation.

Cell surface β1 integrin and MT1-MMP were also investigated by immunofluorescence. β1 integrin expressing cells were better spread, whereas all β1/5 and β1/5 knockdown cells displayed rounder and less-spread morphology, with β1/6 integrin siRNA being most pronounced (Figure 3E). Cell surface β1 integrin localized to the cell periphery while MT1-MMP was more diffusely distributed across the cell but concentrated on the cell surface, and the levels of MT1-MMP appeared to correlate with those of β1 integrin. Reduced cell surface β1 integrin and MT1-MMP was only seen for β1/6 integrin siRNA–treated cells, compared to β1/4 and β1/5, irrespective of Col I treatment (Figure 3F). Additionally, β1 integrin and MT1-MMP levels examined by immunofluorescence corresponded to Western blot determination (Figure 3C, D). Thus, β1/6 integrin siRNA showed the most efficient β1 integrin knockdown, which coincided with the strongest reduction of MT1-MMP.

To confirm that the reduced MMP-2 activation after β1 integrin knockdown was due to reduced MT1-MMP, MCF-7-MT1 cells were transfected with β1/4, β1/5 and β1/6 integrin siRNAs, and subsequently transfected with MT1-MMP. Forced MT1-MMP expression was confirmed (Figure 3G), and the expected suppression of Col I-induced MMP-2 activation by β1/6 integrin siRNA treatment was rescued (Figure 3H). These results confirm a role for β1 integrin in regulating MT1-MMP to facilitate MMP-2 activation in response to Col I stimulation.

### High threshold of β1 Integrin knockdown required to inhibit MMP-2 activation in response to Col I stimulation

**Figure 4:**
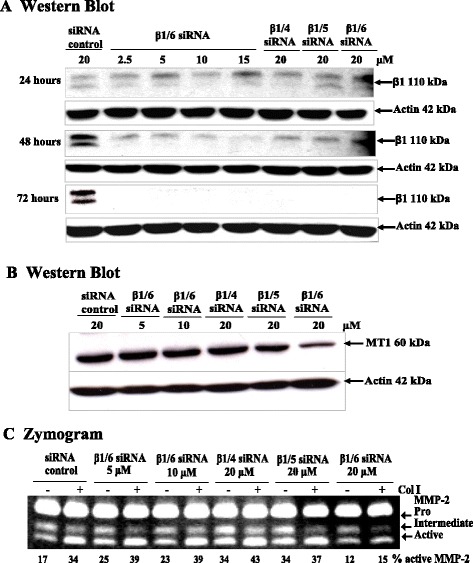
**Dose response effect of β1 integrin siRNA on MMP-2 activation in MCF-7-MT1 cells. (A)**: Cells were plated and transfected with β1 integrin siRNA. Serial dilutions of β1/6 siRNA (2.5, 5, 10, 15 and 20 μM) were used in comparison with the 20 μM concentrations of β1/4 and β1/5 siRNAs. Protein concentrations were determined and equal amounts of lysates were electrophoresed with 10% SDS-PAGE under reducing conditions. Western Blot analysis was used to detect β1 integrin and MT1-MMP levels **(B)**. **(C)**: Serial dilutions of β1/6 siRNA (5, 10, and 20 μM) were used in comparison with the 20 μM concentrations of β1/4 and β1/5 siRNAs. Conditioned medium was collected and effects on MMP-2 activation were examined by zymography.

There are several factors that may influence the efficiency of RNAi in mammalian systems including the choice of the target site of degradation, the transfection method and the turnover rate of the protein. In addition, the mRNA degradation rate is an important factor influencing target gene knockdown by siRNA technology [40,41]. This effect is dependent on the dose response to siRNA concentration. We examined whether β1/4, β1/5 and β1/6 siRNAs had dose-dependent effects on the abrogation of β1 integrin mRNA expression, and whether this was relative to the reduction seen in MMP-2 activation. We hypothesized that the β1/6 siRNA showed a quicker mRNA degradation rate than either β1/4 or β1/5 siRNA. Serial concentrations of β1/6 siRNA (2.5, 5, 10, 15 and 20 μM) were tested. The data showed that at the lowest concentration (2.5 μM), β1/6 siRNA was able to knockdown β1 integrin expression to an extent comparable to the highest concentration (20 μM) of β1/4 or β1/5 siRNA (Figure 4A). The β1 integrin expression was below the threshold of detection 72 hours after transfection with 2.5 and 5 μM of β1/6 siRNA, and this was also seen with β1/4 and β1/5 siRNA at 20 μM. MMP-2 activation reductions were observed only at 20 μM of β1/6 integrin siRNA (Figure 4C), was which relative to reduction of MT1-MMP protein level at this concentration (Figure 4B). These results indicate that the reduction of Col I-induced MMP-2 activation seen only with β1/6 may require the more rapid and complete knock down of β1 integrin seen with this siRNA. A threshold of β1 integrin knockdown that cannot be surpassed by β1/4 or β1/5 siRNAs may exist for the MMP-2 activation effect.

### The suppression of MMP-2 activation by β1/6 siRNA was specific to β1 integrin

**Figure 5:**
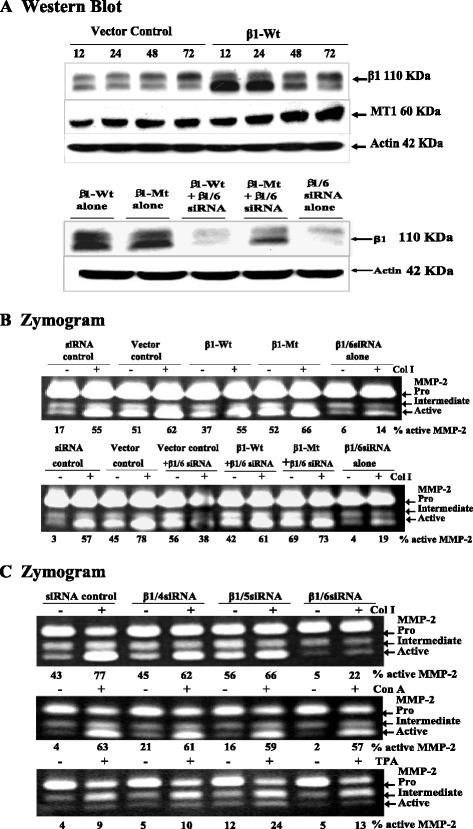
**The target site of β1/6 integrin siRNA was specific to β1 integrin gene.** Site-specific mutagenesis primers were designed around the target site of the β1/6 siRNA. Co-transfections of β1/6 siRNA and either wild type β1 integrin or β1 integrin codon-swapped mutant were performed. **(A)**: Western blot analysis of β1 integrin and MT1-MMP expressions, and the effects on MMP-2 activation were examined by zymography in MCF-7-MT1 cells **(B)**. **(C)**: MCF-7-MT1 cells were treated with Col I (100 μg/ml), Con A (10 μg/ml) or TPA (100nM) after β1/6 siRNA transfection. Conditioned medium was collected and effects on MMP-2 activation were examined by zymography. Semi-quantitative densitometry was performed using the Image J 1.46 program (NIH) and is expressed in percent of area.

Wild-type β1 integrin transfection was successful at increasing β1 integrin expression at all time points, however, strong inductions were observed at 12 and 24 hours after transfection. Consistently, cells over-expressing β1 integrin showed higher MT1-MMP expression than vector control cells at 24, 48 and 72 hours (Figure 5A). The MT1-MMP levels in the vector control cells resembled that seen in β1 integrin knockdown cells without any further transfection (Figure 3C).

Co-transfection of β1 integrin codon-swapped mutant and β1/6 integrin siRNA failed to knockdown β1 integrin, whereas co-transfection of wild-type β1 integrin and β1/6 integrin siRNA (β1/6 integrin siRNA alone) successfully knocked down β1 integrin (Figure 5A). Co-transfection of β1 integrin codon-swapped mutant and β1/6 integrin siRNA rescued the otherwise inhibited MMP-2 activation in both MCF-7-MT1 cells (Figure 5B) and MDA-MB-231 cells (data not shown). These results indicated that the stronger effect of β1/6 integrin siRNA was specific to β1 integrin.

Stimulators of MMP-2 activation that do not function through β1 integrins, such as Con A and TPA, would be expected to remain unaffected by β1 integrin knockdown. As expected, MMP-2 activation by Con A or TPA was not inhibited by β1/6 integrin siRNA treatment (Figure 5C).

## Discussion

The β1-integrins have been linked to tumor progression and the remodeling of extracellular matrix (ECM) associated with this progression [42,43]. Collagen is a major ECM component that plays an important role in maintaining tissue architecture. Several studies point to the necessity of matrix degradation for migration and invasion through interstitial collagen by normal and neoplastic cells, consistent with observations that fibrillar Col I stimulates activation of MMP-2 [17,18,21,22,24,31,44]. Several studies have shown that culturing cells within a gel of Col I stimulates MMP-2 activation through both transcriptional and non-transcriptional enhancement of MT1-MMP [17,18,22,23], and indicated that collagen-binding integrins interact with collagen for this mechanism [19,28,31]. Clustering of integrin β1 was shown to cause increased cell surface MT1-MMP, colocalization of MT1-MMP with integrin, and increased MMP-2 activation [21,28]. In contrast, a recent study indicated that inhibition by integrin β1-targeting siRNA did not affect 3-D collagen-induced cell surface localization of MT1-MMP or MMP-2 activation in mesothelioma cells [31]. These authors concluded that 3-D collagen scaffolding provides a direct and multivalent interaction with MT1-MMP, allowing MMP-2 activation to occur in a cell surface MT1-MMP-dependent manner, rather than a manner regulated by matrix stiffness and integrin β1 function. Our results are inconsistent with these studies, showing an important role of integrin β1 in both the stabilization of MT1-MMP protein levels by collagen, and the increased activation of MMP-2.

In the transcriptional response of Col I-induced MMP-2 activation, Col I can increase MT1-MMP mRNA and protein levels, thereby promoting MMP-2 activation. MCF-7-MT1 is a breast cancer cell line that lacks endogenous MT1-MMP expression. In MCF-7-MT1 cells, which are stably transfected with MT1-MMP, Col I causes activation of MMP-2 despite MT1-MMP being under the control of a heterologous promoter. Furthermore, when these cells were treated with cycloheximide to block new protein synthesis, some reduction in MMP-2 activation was seen, but the Col I response was still observed. Thus, MCF-7-MT1cells provide a model for non-transcriptional regulation of Col I-induced MMP-2 activation [24]. In the non-transcriptional response, Col I blocks MT1-MMP internalization, causing retention of MT1-MMP on the cell surface, and leading to increased MMP-2 activation [24]. Interestingly, the engagement and clustering of β1 integrins on endothelial cells by Col I has been shown to induce a physical interaction between MT1-MMP and β1 integrin, which correlated with an inhibition of MT1-MMP internalization [43]. Moreover, β1 integrins are mainly associated with the 60-kD mature form of MT1-MMP, pointing to an association with active MT1-MMP [43]. Treatment with anti-β1 integrin neutralizing antibodies caused impaired MT1-MMP localization at cell–cell contacts, suggesting that the β1 integrin interaction might be important for MT1-MMP localization.

Col I did not alter total MT1-MMP protein levels, nor did it directly induce MT1-MMP oligomerization. Col I did however redistribute pre-existing MT1-MMP to the cell periphery compared to unstimulated cells that displayed a more diffuse staining pattern. In addition, Col I blocked the internalization of MT1-MMP in a dynamin-dependent manner via clathrin-coated pit mediated endocytosis [24]. In the current study, we found that abrogation of β1 integrin blocked up-regulation of MT1-MMP activity by Col I; β1 integrin knockdown reduced overall MT1-MMP protein levels. These findings support previous observations that a physical interaction between MT1-MMP and β1 integrin correlates with an inhibition of MT1-MMP internalization in the presence of Col I [45].

MMP-2 activation and fibrillar collagen degradation are thought to be important functions of MT1-MMP. Both of these activities uniquely require MT1-MMP to be in a dimeric form on the cell surface, and inhibition of dimerisation effectively inhibits these activities [46,47]. The β1/6 integrin siRNA not only caused the most efficient knockdown of β1 integrin, but also most highly affected down-regulation of MT1-MMP. This reduction of MT1-MMP in response to Col I in β1/6 integrin siRNA knockdown cells was neutralized by MT1-MMP overexpression, which caused a reciprocal strong induction of β1 integrin at 12 and 24 hours, similar to the time frame in which the transiently transfected MT1-MMP expression takes place (data not shown). This subsequently enhanced the Col I-induced MMP-2 activation by MT1-MMP. These data complement previous studies indicating that down-regulation of β1 integrin reduces expression of MT1-MMP [48]. In addition, MT1-MMP cooperates with β1-integrin during the migration of endothelial cells on various ECMs [16], and was found to colocalize with β1-integrin in actin-rich, “collagenolysis-free” leading edges of migrating fibrosarcoma and breast carcinoma cells grown on a 3D collagen matrix [49]. In our system, the suppression of MMP-2 activation by β1/6 integrin siRNA was specific to Col I stimulation, and did not affect MMP-2 activation stimulated by Con A or TPA. Con A likely works by clustering proteins with terminal sialic acid residues, however it seems not to include β1 integrin [50].

Although we identified that abrogation of β1 integrin caused reduced Col I-induced MMP-2 activation, we observed that a very high threshold of inhibition was required, more so than needed for other β1 integrin functionalities such as substrate adhesion and collagen gel contraction. Collagen gel contraction occurs through a process of reorganization of the collagen fibrils, and is used as an *in vitro* model of cell-mediated tissue remodeling, including wound contraction and maintenance of tissue homeostasis [51]. Several studies have demonstrated a role for β1 integrins, in particular α1β1 and α2β1 integrins, in mediating collagen gel contraction by fibroblasts and osteoblasts [52,53]. The three β1 integrin siRNAs tested showed good knockdown of β1 integrin, but only β1/6 caused a reduction in Col I-stimulated MMP-2 activation. β1 integrin siRNAs showed knockdown of β1 integrin expression after 24 hours of transfection, however, complete knockdown of β1 integrin was only observed at 72 hours. Importantly, the reduction in MMP-2 activation occurred at 72 hours, commensurate with the most complete abrogation of β1 integrin. The only β1 integrin siRNA that blocked Col I-induced MMP-2-activation was more potent in terms of both dose response and time course of inhibition than the other 2 tested, suggesting that an important threshold may exist for the level of β1 integrin required to mediate Col I-stimulated MMP-2-activation. It is possible that the mesothelioma study [29] did not achieve sufficient abrogation of β1 integrin to reduce MMP-2 activation at 48 hours after transfection integrin β1-targeting siRNA where β1 integrin is not complete knockdown.

MT1-MMP expression is induced in fibroblast and endothelial cells by culture in three-dimensional collagen matrixes [19,23]. Therefore, signaling pathways initiated through collagen binding β1 integrin may regulate MT1-MMP expression. The interaction between β1 integrin and MT1-MMP blocks MT1-MMP internalization and causes increased MMP-2 activation. It is now widely accepted that MT1-MMP is regulated by and/or associated with integrin/FAK/Src/P13C as a pathway to promote pericellular proteolysis and invasion in 3-dimensional cultures [54], in part due to focal adhesion turnover [55,56]. FAK-mediated src phosphorylation of endophilin A2 inhibits endocytosis of MT1-MMP and promotes ECM degradation [57]. In addition, osteopontin, an ECM protein, is able to interact with αVβ3 integrin to enhance MT1-MMP expression, stimulate MMP-2 activation, and induce cell migration and invasion in murine melanoma cells [58]. Several lines of evidence suggest that integrin αVβ3 binds MMP-2 and acts as a receptor for surface-localized MMP activity. These studies demonstrated that αVβ3 and MMP-2 were co-localized on the surface of invasive angiogenic vascular cells, and melanoma or cervical cancer cells [16,59,60]. In our MCF-7-MT1 or MDA-MB-231 cells, overexpression of β1 integrin did not stimulate MMP-2 activation despite an apparent increase in the steady state levels of total MT1-MMP. These observations support a complex biphasic model whereby Col I-ligated β1-integrin acts both locally and via signaling to upregulate pericellular proteolysis.

## Conclusions

This study confirmed the role of β1 integrin in the MMP-2 activation response of breast cancer cells to Col I. This work has demonstrated that abrogation of β1 integrin caused down-regulation of MT1-MMP, α2 and α3 integrins, and reduced MMP-2 activation in response to Col I. The reduction in MMP-2 activation required the most complete abrogation of β1 integrin, suggesting that an important threshold may exist for the level of β1 integrin required to mediate Col I-stimulated MMP-2-activation. This may provide insights into the mechanism involved in the reciprocal regulation of integrin and MMPs on the surface of tumour cells.

## Abbreviations

Col I: Type I collagen
Con A: Concanavalin A
ECM: Extracellular matrix
FN: Fibronectin
LM: Laminin-1
MMP: Matrix metalloproteinase
MT-MMP: Membrane-type matrix metalloproteinase
siRNA: Short interfering RNA
TIMP: Tissue inhibitor of metalloproteinases
TPA: 12-O-tetradecanoylphorbol-13-acetate
VN: Vitronectin

## References

[1] Humphries MJ (2000). Integrin structure. Biochem Soc Trans.

[2] Schatzmann F, Marlow R, Streuli CH (2003). Integrin signaling and mammary cell function. J Mammary Gland Biol Neoplasia.

[3] Elliott BE, Ekblom P, Pross H, Niemann A, Rubin K (1994). Anti-beta 1 integrin IgG inhibits pulmonary macrometastasis and the size of micrometastases from a murine mammary carcinoma. Cell Adhes Commun.

[4] Fujita S, Watanabe M, Kubota T, Teramoto T, Kitajima M (1995). Alteration of expression in integrin beta 1-subunit correlates with invasion and metastasis in colorectal cancer. Cancer Lett.

[5] Cordes N, Park CC (2007). beta1 integrin as a molecular therapeutic target. Int J Radiat Biol.

[6] Lynch CC, Matrisian LM (2002). Matrix metalloproteinases in tumor-host cell communication. Differentiation.

[7] Overall CM, Kleifeld O (2006). Tumour microenvironment - opinion: validating matrix metalloproteinases as drug targets and anti-targets for cancer therapy. Nat Rev Cancer.

[8] Fingleton B (2006). Matrix metalloproteinases: roles in cancer and metastasis. Front Biosci.

[9] Egeblad M, Werb Z (2002). New functions for the matrix metalloproteinases in cancer progression. Nat Rev Cancer.

[10] Noel AC, Polette M, Lewalle JM, Munaut C, Emonard HP, Birembaut P, Foidart JM (1994). Coordinate enhancement of gelatinase A mRNA and activity levels in human fibroblasts in response to breast-adenocarcinoma cells. Int J Cancer.

[11] Dalberg K, Eriksson E, Enberg U, Kjellman M, Backdahl M (2000). Gelatinase A, membrane type 1 matrix metalloproteinase, and extracellular matrix metalloproteinase inducer mRNA expression: correlation with invasive growth of breast cancer. World J Surg.

[12] Visse R, Nagase H (2003). Matrix metalloproteinases and tissue inhibitors of metalloproteinases: structure, function, and biochemistry. Circ Res.

[13] Itoh Y, Seiki M (2004). MT1-MMP: an enzyme with multidimensional regulation. Trends Biochem Sci.

[14] Sato H, Takino T, Okada Y, Cao J, Shinagawa A, Yamamoto E, Seiki M (1994). A matrix metalloproteinase expressed on the surface of invasive tumour cells. Nature.

[15] Sato H, Takino T, Kinoshita T, Imai K, Okada Y, Stetler Stevenson WG, Seiki M (1996). Cell surface binding and activation of gelatinase A induced by expression of membrane-type-1-matrix metalloproteinase (MT1-MMP). FEBS Lett.

[16] Strongin AY, Collier I, Bannikov G, Marmer BL, Grant GA, Goldberg GI (1995). Mechanism of cell surface activation of 72-kda type IV collagenase. Isolation of the activated form of the membrane metalloprotease. J Biol Chem.

[17] Azzam HS, Thompson EW (1992). Collagen-induced activation of the M(r) 72,000 type IV collagenase in normal and malignant human fibroblastoid cells. Cancer Res.

[18] Thompson EW, Yu M, Bueno J, Jin L, Maiti SN, Palao-Marco FL, Pulyaeva H, Tamborlane JW, Tirgari R, Wapnir I, Azzam H (1994). Collagen induced MMP-2 activation in human breast cancer. Breast Cancer Res Treat.

[19] Haas TL, Davis SJ, Madri JA (1998). Three-dimensional type I collagen lattices induce coordinate expression of matrix metalloproteinases MT1-MMP and MMP-2 in microvascular endothelial cells. J Biol Chem.

[20] Deryugina EI, Bourdon MA, Reisfeld RA, Strongin A (1998). Remodeling of collagen matrix by human tumor cells requires activation and cell surface association of matrix metalloproteinase-2. Cancer Res.

[21] Ellerbroek SM, Wu YI, Overall CM, Stack MS (2001). Functional interplay between type I collagen and cell surface matrix metalloproteinase activity. J Biol Chem.

[22] Kim IY, Jeong SJ, Kim ES, Kim SH, Moon A (2007). Type I collagen-induced pro-MMP-2 activation is differentially regulated by H-Ras and N-Ras in human breast epithelial cells. J Biochem Mol Biol.

[23] Gilles C, Polette M, Seiki M, Birembaut P, Thompson EW (1997). Implication of collagen type I-induced membrane-type 1-matrix metalloproteinase expression and matrix metalloproteinase-2 activation in the metastatic progression of breast carcinoma. LabInvest.

[24] Lafleur MA, Mercuri FA, Ruangpanit N, Seiki M, Sato H, Thompson EW (2006). Type I collagen abrogates the clathrin-mediated internalization of membrane type 1 matrix metalloproteinase (MT1-MMP) via the MT1-MMP hemopexin domain. J Biol Chem.

[25] Prockop DJ, Kivirikko KI (1995). Collagens: molecular biology, diseases, and potentials for therapy. Annu Rev Biochem.

[26] Shoulders MD, Raines RT (2009). Collagen structure and stability. Annu Rev Biochem.

[27] Jokinen J, Dadu E, Nykvist P, Kapyla J, White DJ, Ivaska J, Vehvilainen P, Reunanen H, Larjava H, Hakkinen L, Heino J (2004). Integrin-mediated cell adhesion to type I collagen fibrils. J Biol Chem.

[28] Ellerbroek SM, Fishman DA, Kearns AS, Bafetti LM, Stack MS (1999). Ovarian carcinoma regulation of matrix metalloproteinase-2 and membrane type 1 matrix metalloproteinase through beta1 integrin. Cancer Res.

[29] Tang Y, Rowe RG, Botvinick EL, Kurup A, Putnam AJ, Seiki M, Weaver VM, Keller ET, Goldstein S, Dai J, Begun D, Saunders T, Weiss SJ (2013). MT1-MMP-dependent control of skeletal stem cell commitment via a β1-integrin/YAP/TAZ signaling axis. Dev Cell.

[30] Mori H, Lo AT, Inman JL, Alcaraz J, Ghajar CM, Mott JD, Nelson CM, Chen CS, Zhang H, Bascom JL, Seiki M, Bissell MJ (2013). Transmembrane/cytoplasmic, rather than catalytic, domains of Mmp14 signal to MAPK activation and mammary branching morphogenesis via binding to integrin β1. Development.

[31] Sakai K, Nakamura T, Suzuki Y, Imizu T, Matsumoto K (2011). 3-D collagen-dependent cell surface expression of MT1-MMP and MMP-2 activation regardless of integrin beta1 function and matrix stiffness. Biochem Biophys Res Commun.

[32] Pulyaeva H, Bueno J, Polette M, Birembaut P, Sato H, Seiki M, Thompson EW (1997). MT1-MMP correlates with MMP-2 activation potential seen after epithelial to mesenchymal transition in human breast carcinoma cells. Clin Exp Metastasis.

[33] Gilles C, Bassuk JA, Pulyaeva H, Sage EH, Foidart JM, Thompson EW (1998). SPARC/osteonectin induces matrix metalloproteinase 2 activation in human breast cancer cell lines. Cancer Res.

[34] Lafleur MA, Drew AF, de Sousa EL, Blick T, Bills M, Walker EC, Williams ED, Waltham M, Thompson EW (2005). Upregulation of matrix metalloproteinases (MMPs) in breast cancer xenografts: a major induction of stromal MMP-13. Int J Cancer.

[35] Tulla M, Pentikainen OT, Viitasalo T, Kapyla J, Impola U, Nykvist P, Nissinen L, Johnson MS, Heino J (2001). Selective binding of collagen subtypes by integrin alpha 1I, alpha 2I, and alpha 10I domains. J Biol Chem.

[36] Hynes RO (2002). Integrins: bidirectional, allosteric signaling machines. Cell.

[37] Green LJ, Mould AP, Humphries MJ (1998). The integrin beta subunit. Int J Biochem Cell Biol.

[38] Holmbeck K, Bianco P, Caterina J, Yamada S, Kromer M, Kuznetsov SA, Mankani M, Robey PG, Poole AR, Pidoux I, Ward JM, Birkedal-Hansen H (1999). MT1-MMP-deficient mice develop dwarfism, osteopenia, arthritis, and connective tissue disease due to inadequate collagen turnover. Cell.

[39] Ruangpanit N, Chan D, Holmbeck K, Birkedal-Hansen H, Polarek J, Yang C, Bateman JF, Thompson EW (2001). Gelatinase A (MMP-2) activation by skin fibroblasts: dependence on MT1-MMP expression and fibrillar collagen form. Matrix Biol.

[40] Kim NV (2003). RNA interference in functional genomics and medicine. J Korean Med Sci.

[41] Lipardi C, Wei Q, Paterson BM (2001). RNAi as random degradative PCR. siRNA primers convert mRNA into dsRNA that are degraded to generate new siRNAs. Cell.

[42] Guo W, Giancotti FG (2004). Integrin signalling during tumour progression. Nat Rev Mol Cell Biol.

[43] Larsen M, Artym VV, Green JA, Yamada KM (2006). The matrix reorganized: extracellular matrix remodeling and integrin signaling. Curr Opin Cell Biol.

[44] Ivaska J, Reunanen H, Westermarck J, Koivisto L, Kahari VM, Heino J (1999). Integrin alpha2beta1 mediates isoform-specific activation of p38 and upregulation of collagen gene transcription by a mechanism involving the alpha2 cytoplasmic tail. J Cell Biol.

[45] Galvez BG, Matias-Roman S, Yanez-Mo M, Sanchez-Madrid F, Arroyo AG (2002). ECM regulates MT1-MMP localization with beta1 or alphavbeta3 integrins at distinct cell compartments modulating its internalization and activity on human endothelial cells. J Cell Biol.

[46] Lehti K, Lohi J, Juntunen MM, Pei D, Keski-Oja J (2002). Oligomerization through hemopexin and cytoplasmic domains regulates the activity and turnover of membrane-type 1 matrix metalloproteinase. J Biol Chem.

[47] Itoh Y, Ito N, Nagase H, Evans RD, Bird SA, Seiki M (2006). Cell surface collagenolysis requires homodimerization of the membrane-bound collagenase MT1-MMP. Mol Biol Cell.

[48] Sameni M, Dosescu J, Yamada KM, Sloane BF, Cavallo-Medved D (2008). Functional live-cell imaging demonstrates that beta1-integrin promotes type IV collagen degradation by breast and prostate cancer cells. Mol Imaging.

[49] Wolf K, Wu YI, Liu Y, Geiger J, Tam E, Overall C, Stack MS, Friedl P (2007). Multi-step pericellular proteolysis controls the transition from individual to collective cancer cell invasion. Nat Cell Biol.

[50] Yu M, Sato H, Seiki M, Thompson EW (1995). Complex regulation of membrane-type matrix metalloproteinase expression and matrix metalloproteinase-2 activation by concanavalin A in MDA-MB-231 human breast cancer cells. Cancer Res.

[51] Finesmith TH, Broadley KN, Davidson JM (1990). Fibroblasts from wounds of different stages of repair vary in their ability to contract a collagen gel in response to growth factors. J Cell Physiol.

[52] Gullberg D, Tingstrom A, Thuresson AC, Olsson L, Terracio L, Borg TK, Rubin K (1990). Beta 1 integrin-mediated collagen gel contraction is stimulated by PDGF. Exp Cell Res.

[53] Carver W, Molano I, Reaves TA, Borg TK, Terracio L (1995). Role of the alpha 1 beta 1 integrin complex in collagen gel contraction in vitro by fibroblasts. J Cell Physiol.

[54] Takino T, Tsuge H, Ozawa T, Sato H (2010). MT1-MMP promotes cell growth and ERK activation through c-Src and paxillin in three-dimensional collagen matrix. Biochem Biophys Res Commun.

[55] Takino T, Guo L, Domoto T, Sato H (2013). MT1-MMP prevents growth inhibition by three dimensional fibronectin matrix. Biochem Biophys Res Commun.

[56] Stehbens SJ, Paszek M, Pemble H, Ettinger A, Gierke S, Wittmann T (2014). CLASPs link focal-adhesion-associated microtubule capture to localized exocytosis and adhesion site turnover. Nat Cell Biol.

[57] Wu X, Gan B, Yoo Y, Guan JL (2005). FAK-mediated src phosphorylation of endophilin A2 inhibits endocytosis of MT1-MMP and promotes ECM degradation. Dev Cell.

[58] Phillip S, Bulbule A, Kundu GC (2001). Osteopontin stimulates tumor growth and activation of promatrix metalloproteinase-2 through nuclear factor-kappa B-mediated induction of membrane type I matrix metalloproteinase in murine melanoma cells. J Biol Chem.

[59] Deryugina EI, Ratnikov B, Postnova TI, DiScipio R, Smith JW, Strongin AY (2001). MT1-MMP initiates activation of pro-MMP-2 and integrin alphavbeta3 promotes maturation of MMP-2 in breast carcinoma cells. Exp Cell Res.

[60] Chattopadhyay N, Aparna M, Frei E, Chatterjee A (2001). Human cervical tumor cell (SiHa) surface αvβ3 integrin receptor has associated matrix metalloproteinase (MMP-2) activity. J Cancer Res Clin Oncol.

